# Baroreflex sensitivity during rest and pressor challenges in obstructive sleep apnea patients with and without CPAP^☆^

**DOI:** 10.1016/j.sleep.2022.05.846

**Published:** 2022-06-04

**Authors:** Amrita Pal, Fernando Martinez, Roopsha Chatterjee, Ravi S. Aysola, Ronald M. Harper, Vaughan G. Macefield, Luke A. Henderson, Paul M. Macey

**Affiliations:** a UCLA School of Nursing, University of California Los Angeles, Los Angeles, CA, USA; b Division of Pulmonary and Critical Care, Department of Medicine, David Geffen School of Medicine at UCLA, University of California Los Angeles, Los Angeles, CA, USA; c Neurobiology, David Geffen School of Medicine at UCLA, University of California Los Angeles, Los Angeles, CA, USA; d Baker Heart and Diabetes Institute, Melbourne, and Department of Anatomy and Physiology, School of Biomedical Sciences, The University of Melbourne, USA; e Department of Anatomy and Histology, Sydney Medical School, University of Sydney, Sydney, Australia

**Keywords:** Autonomic, Cardiovascular, Blood pressure variability

## Abstract

**Introduction:**

Obstructive sleep apnea (OSA) increases sympathetic vasoconstrictor drive and reduces baroreflex sensitivity (BRS), the degree to which blood pressure changes modify cardiac output. Whether nighttime continuous positive airway pressure (CPAP) corrects BRS in the awake state in OSA remains unclear. We assessed spontaneous BRS using non-invasive continuous BP and ECG recordings at rest and during handgrip and Valsalva challenges, maneuvers that increase vasoconstrictor drive with progressively higher BP, in untreated OSA (unOSA), CPAP-treated OSA (cpOSA) and healthy (CON) participants.

**Methods:**

In a cross-sectional study of 104 participants, 34 unOSA (age mean±std, 50.6±14.1years; Respiratory Event Index [REI] 21.0±15.3 events/hour; 22male), 31 cpOSA (49.6±14.5years; REI 23.0±14.2 events/hour; 22male; self-report 4+hours/night,5+days/week,6months), and 39 CON (42.2±15.0years; 17male), we calculated BRS at rest and during handgrip and Valsalva. Additionally, we correlated BP variability (BPV) with BRS during these protocols.

**Results:**

BRS in unOSA, cpOSA and CON was, respectively (mean±sdv in ms/mmHg), at rest: 14.8±11.8, 15.8±17.0, 16.1±11.3; during handgrip 13.3±7.6, 12.7±8.4, 16.4±8.7; and during Valsalva 12.7±8.0, 11.5±6.6, 15.1±8.9. BRS was lower in cpOSA than CON for handgrip (*p*=0.04) and Valsalva (*p*=0.03). BRS was negatively correlated with BPV in unOSA during Valsalva and handgrip for cpOSA, both R=−0.4 (*p*=0.02). BRS was negatively correlated with OSA severity (levels: none, mild, moderate, severe) at R=−0.2 (*p*=0.04,n=104).

**Conclusions:**

As expected, BRS was lower and BPV higher in OSA during the pressor challenges, and disease severity negatively correlated with BRS. In this cross-sectional study, both CPAP-treated (self-reported) and untreated OSA showed reduced BRS, leaving open whether within-person CPAP improves BRS.

## 1. Introduction

Autonomic regulation is altered in obstructive sleep apnea (OSA) during both sleep [1] and wakefulness [2,3]. For example, sympathetic outflow is high as reflected in greater muscle sympathetic nerve activity (MSNA) [1], which, over time, often leads to hypertension [4]. The high blood pressure accompanying OSA is commonly treatment-resistant to both anti-hypertensive medications and continuous positive airway pressure (CPAP) [5]. Regular use of CPAP does normalize MSNA, suggesting that other impairments, perhaps vascular remodeling or long-term neural injury to cardiac regulatory areas of the brain, contribute to the hypertension. Insight into these impairments may be gained from understanding autonomic mechanisms in treated and untreated OSA, in particular, the baroreflex. The baroreflex operates to maintain blood pressure (BP) relatively constant via changes in cardiac output and total peripheral resistance, and can be assessed by measurement of baroreflex sensitivity (BRS) [1,6,7]. Extensive structural injury occurs in the brain in OSA [8-10], including the brainstem where the essential circuitry responsible for the baroreflex is located [11-14]. Moreover, in OSA there are significant changes in functional activity related to BP control in the brain, as revealed through MSNA-coupled fMRI [15]. However, there is some evidence that the structural and functional changes may be partially reversed following 6 months of CPAP [16,17]. Therefore, we tested the hypothesis that baroreflex function is unimpaired in OSA patients using CPAP.

The baroreflex acts to maintain blood pressure within a narrow homeostatic range, although the set-point changes depending on physiological demands, such as during exercise [18]. It operates as a negative-feedback system in which stretch-sensitive baroreceptors located in the carotid sinus and aortic arch relay sensory information via the glossopharyngeal and vagus nerves to the nucleus tractus solitarius (NTS) in the medulla. From there, excitatory synapses are made with the nucleus ambiguus (NA) and the dorsal motor nucleus of the vagus (DMNV), which send excitatory projections to the parasympathetic ganglia of the heart. Those neurons within the ganglia can reduce the rate and force of cardiac contraction. Excitatory neurons in NTS also send excitatory projections to the caudal ventrolateral medulla (CVLM), which makes inhibitory synapses with the bilateral rostral ventrolateral medulla (RVLM), the final output nuclei for generating bursts of MSNA [19,20]. The baroreflex is often impaired in cardiovascular diseases [21], including OSA [22], and in neurological disorders [12]. A failure at any level of the reflex arc–baroreflex afferent/central efferent pathway could affect baroreflex function, resulting in exaggerated or depressed BRS [23].

Depressed BRS appears in untreated OSA patients [24], but there is evidence CPAP treatment improves baroreflex control during sleep in OSA [25]. Whether this improvement extends to the awake state is unclear; during wakefulness, OSA patients show augmented sympathetic activity as reflected by increased MSNA [26,27]. Additionally, during wakefulness, OSA patients show a decreased sympathetic component of the BRS as measured by how effectively the baroreflex buffers beat-to-beat changes in blood pressure through modulation of MSNA. OSA also shows decreased BRS as measured by the sensitivity of baroreflex control of the heart assessed using the relationship between R-R interval and systolic blood pressure (sometimes called “cardiac” BRS) [28]. However, other studies have found that although OSA patients show depressed sympathetic components of the BRS, they did not differ from healthy people in cardiac BRS [29]. The focus here is on cardiac BRS, which we refer to just as BRS. As an aside, depressed BRS is often associated with higher severity of OSA [30], but mild-to-moderate OSA does not always show depressed BRS [6]. Regardless, when impaired BRS is present, it could presumably contribute to the development of cardiovascular disease independently of what happens during sleep. Long-term CPAP treatment has been thought to improve baroreflex dysfunction in OSA partly by resolving nocturnal intermittent hypoxia [31]. MSNA during waking is reduced to healthy levels with continual CPAP treatment for 6 months [32], an improvement that may arise from restoration of brainstem autonomic structures and function [16,33]; however, it is unclear if treatment resolves baroreflex dysfunction in OSA during wakefulness.

The baroreflex is recruited with any pressor challenge, such as handgrip or Valsalva [34-36], including in OSA [37]. BRS has been evaluated during rest and in response to exercise training using continuous BP and ECG recordings in OSA participants [38]. During a standard handgrip challenge, after 15–30 s of isometric gripping, BP and heart rate (HR) increase [35,36]. During a Valsalva maneuver, HR and BP increase after 10–20 s of forced expiration and, upon release, change with a distinct rapid fluctuation (Phase III) and longer recovery (Phases IV); these responses differ in OSA compared with healthy people [39,40]. These well-characterized BP changes elicited with standard challenges provide an opportunity to test the BRS.

Low BRS normally leads to high BP variability (BPV), since the weaker regulation results in exaggerated BP fluctuations [41,42]. This relationship is consistent with observations in OSA of high BPV [2,43] and low BRS [1,28]. Thus, in OSA any reduced BRS during wakefulness should be associated with an increase in BPV.

One further consideration is sex differences. Previously, we showed a higher BPV in females with OSA [2], which could result from a more depressed BRS in women as opposed to men with OSA [44]. Sex differences were observed in the insular cortex in response to handgrip [45]; however, no OSA-specific difference was observed in the functional response of the insula to handgrip irrespective of sex [46]. Given that the early increase in HR during handgrip is believed to be due to withdrawal of cardiac vagal drive, this parasympathetic withdrawal contribution to HRV in response to a standardized handgrip challenge has been proposed to remain the same in OSA as in healthy people irrespective of sex [46]. However, the insula responses to the Valsalva showed OSA-specific effects in only in females, who showed greater right anterior dominance [39]. These variable findings reinforce the importance of considering sex differences when assessing autonomic function in OSA.

The objective here was to quantify BRS during wakefulness in untreated OSA (unOSA), CPAP-treated OSA (cpOSA) and healthy controls (CON) during rest, handgrip and Valsalva, while accounting for sex. Additionally, we aimed to quantify the association between BRS and BRV. As a first step, we performed a study with a cross-sectional design. We hypothesized BRS would show group differences between each combination of unOSA, cpOSA, and CON. We also hypothesized that BPV would be inversely related to BRS.

## 2. Methods

### Design:

We performed a cross-sectional study of three groups based on a convenience sample.

### Recruitment and screening:

Recruitment fliers were posted on the UCLA campus and in the UCLA Sleep Disorders Center, and on online sites advertising UCLA research studies. Fliers for untreated OSA participants targeted people with confirmed or suspected OSA, fliers for treated OSA targeted people using CPAP, and fliers for control participants targeted healthy people. Participants in the OSA groups had their sleep disorder diagnosed by the UCLA Sleep Disorders Center via an at-home test.

After initiating contact, potential participants underwent a phone screening followed by a more comprehensive online survey to assess eligibility. Phone screening included questions about diagnosed sleep disorders, sleep complaints, mental health disorders, or snoring. Participants who passed the phone screening completed an online questionnaire that included questions about medical history, sleep disorders, sleep complaints, menopausal status, and daytime sleepiness. Participants reporting daytime sleepiness or other sleep complaints were given a two–night home sleep apnea testing (HSAT) through the UCLA Sleep Disorders Center to screen for OSA and other sleep disorders. Exclusion criteria for all participants included sleep disorders other than OSA, major illness or head injury, stroke, major cardiovascular disease, current tobacco use, recent (< 3 months) use of psychotropic medications, recent use of cardiovascular medications with major autonomic influences (including angiotensin converting enzyme inhibitors, angiotensin receptor blockers, beta blockers), and diagnosed mental health disorder (other than anxiety or unipolar depressive conditions). Hypertension was reported in ten people (four cpOSA, four unOSA and two CON).

### Sample:

We enrolled 34 unOSA, 31 cpOSA and 39 CON participants (Table 1). There were significant age differences between groups, with CON younger than OSA. There were 15 unOSA and 11 cpOSA participants diagnosed with mild OSA, 11 unOSA and 8 cpOSA participants diagnosed with moderate OSA, and, 8 unOSA and 12 cpOSA participants diagnosed with severe OSA. Some of these participants were studied previously [2]. CPAP usage was self-reported and adherence of 4+ hours/night at least five days/week for at-least 6 months was set as the inclusion criteria for the cpOSA group.

### Ethics:

All procedures were approved by the UCLA Institutional Review Board. All participants provided written informed consent.

### Sleep studies:

Participants without a recent diagnosis (<6 months) or with suspected OSA were referred for an HSAT with an ARES^™^ device (https://sleepmedinc.com/sleep-solutions/ares-home-testing/) [47]. ARES^™^ has the electrodes frontopolar (FP)1 and FP2 for deriving EEG, electro-oculogram (EOG) and electro-myogram (EMG), but it does not qualify for the American Academy of Sleep Medicine (AASM) definition of HSAT sleep vs wake states; the device allows for calculating the respiratory event index (REI) as opposed to the apnea–hypopnea index (AHI). The ARES device captures airflow using a nasal cannula and pressure transducer. Apnea was defined as a cessation (> 90% reduction) in flow for ≥ 10 seconds, hypopnea ≥ 50% reduction in flow for ≥ 10 seconds; the criterion for REI apneas and hypopneas was 4% desaturation [48]. The scoring assigned to participants was based on the average over the single night with the longest valid recording time. The unOSA participants were not using CPAP or any other treatment for the sleep disorder, although since the sleep studies were not performed at the same time as data collection (mean ± SD time difference between physiology and sleep study = 25.1 ± 26.6 days), the group included people who had been offered but did not use CPAP.

Thirteen people in the control group had HSAT sleep studies and one person was diagnosed with OSA, and categorized as unOSA. In the remaining 12 control participants, the REI mean ± SD was 0.5±0.8 events/hr, minimum oxygen saturation was 89±6% and average oxygen saturation was 96±2%.

### Protocol:

**Fig. 1.**
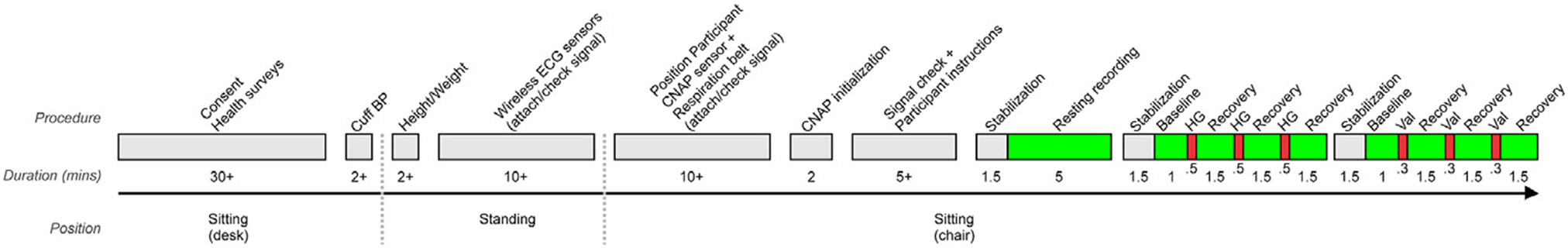
Protocol timeline. Protocol timeline showing procedure (top), duration in minutes (middle) and participant position (bottom). Colored areas show data used for analysis, with green indicating rest or baseline and recovery, and red indicating tasks. BP = blood pressure; CNAP = continuous non-invasive arterial pressure; ECG = electrocardiogram; HG= Handgrip; Val= Valsalva..

Participants were asked to avoid caffeine or other stimulants 24 h beforehand, and to avoid eating before their visit if possible or limit their food intake to a light meal. Visits were scheduled mid–morning (9:30 a.m. earliest) to early evening (6:30 p.m. latest start). We measured participants' height and weight for BMI calculation, and recorded resting BP (Omron 3 series BP monitor, Kyoto, Japan). We obtained ECG and beat-to-beat continuous non-invasive arterial pressure (CNAP Monitor 500, CNSys-tems, Berlin, Germany). Automated finger cuffs and brachial BP cuffs were placed on the participant's left middle and index fingers and their left upper arm at the brachial artery, respectively. Three ECG electrodes were placed on the participant's torso according to standard placements. Continuous data were collected with BIOPAC's MP150 system (BIOPAC Systems Inc, Goleta, CA, USA). The CNAP monitor is based on pressure at the finger, and is calibrated with an automatically-obtained brachial cuff measure (“CNAP initialization” in Fig. 1) [2]. At initialization, the CNAP signal is matched to the brachial cuff values. We used the CNAP v3.5 protocol with adult default settings (https://www.biopac.com/wp&ndash;content/uploads/nibp100d_cnap_monitor.pdf) [49]. The filter setting on the CNAP acquisition was 1 kHz low pass. All data were sampled at 1 kHz.

Fig. 1 demonstrates the data acquisition timeline. The rest protocol consisted of 5 min’ seated resting physiologic data, following a 90 s stabilization period. The handgrip protocol involved three challenge periods at 80% of maximum grip strength using an electric handgrip dynamometer. After 60 s of baseline stabilization, there were 30 s of handgrip followed by 90 s of recovery, repeated twice for a total of three times (60 s baseline, 90 s recovery, 80% maximum strength), resulting in a 7 min protocol. Maximum grip strength was calculated earlier in the session based on the average of three attempts to grip as hard as possible. Participants viewed a real-time display of their grip strength relative to the target, and were instructed to maintain grip levels just above the target. The Valsalva protocol involved three challenge periods with target of 30 mmHg expiratory pressure. After 60 s of baseline stabilization, there were 16 s of Valsalva followed by 90 s of recovery, repeated for a total of three times. A visual display was used to guide participants to maintain the target pressure.

### Analysis–BRS calculation:

**Fig. 2.**
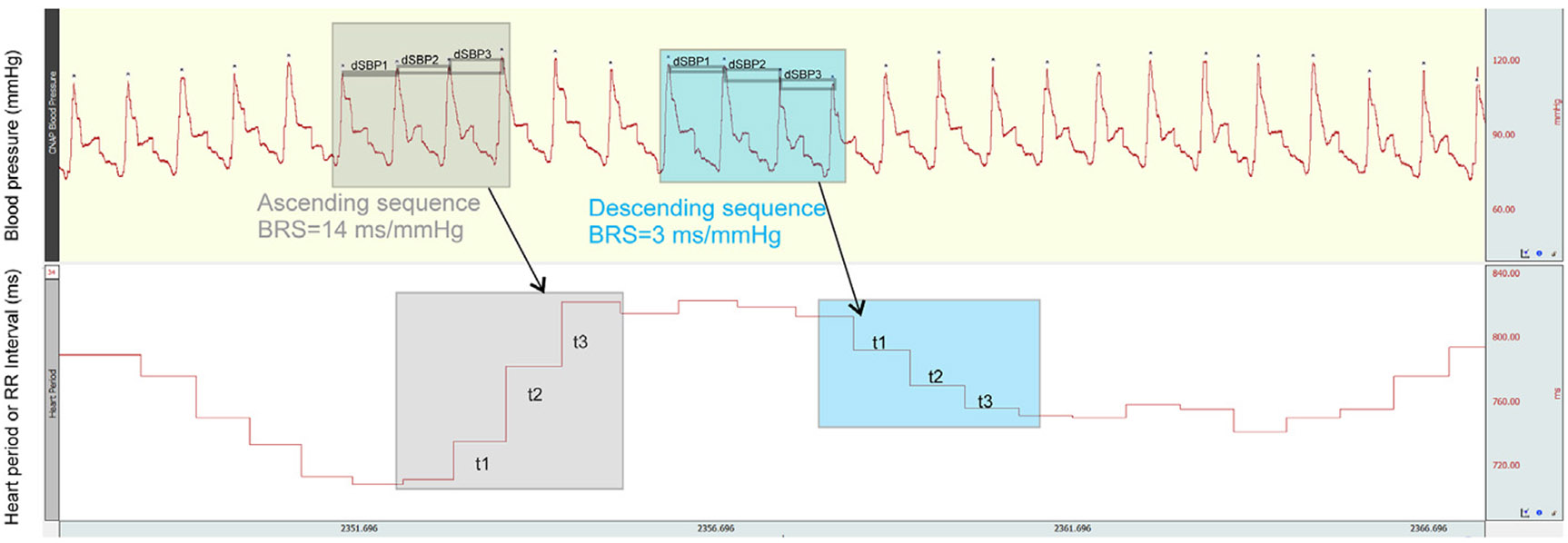
Sample traces illustrating baroreflex sensitivity (BRS) calculation. Illustration of the BRS sequence analysis method in Acqknowledge 5.0. Ascending and descending sequences are defined as at least three cycles showing changes in blood pressure of at least 0.5 mmHg and changes in RR interval of at least 1 ms. For each systolic blood pressure (SBP)-to-SBP change (dSBP), the corresponding change in RR interval (t) is extracted, and the BRS is calculated as the absolute value of the regression coefficient of the change in t over dSBP over the sequence.

An index of baroreflex sensitivity (BRS) is the millisecond increase in inter-beat RR-interval per mmHg change in BP [34]. The RR-interval was calculated from R-waves of the ECG using “Acqknowledge” 5.0 software with manual verification of all peaks (BIOPAC Systems Inc, Goleta, CA, USA). Spontaneous baroreflex sensitivity (BRS) in the time domain can be measured from sequences of beats in which the absolute changes between consecutive ascending or descending systolic pressure values are greater than a given threshold; the corresponding ECG waves allow calculation of the corresponding HR changes in th sequence [21,50]. Fig. 2 demonstrates the BRS calculation for an ascending and descending sequence. The change in R-R intervals occurred about 1 ms after at least 0.5 mmHg change in systolic BP (* in Fig. 2) in at least three consecutive cycles, as per the BRS sequence method implemented in Acqknowledge 5.0. For each SBP-to-SBP change (dSBP), the corresponding change in RR interval (t) is extracted, and the BRS is calculated as the regression coefficient of change in t over change in SBP in an ascending or descending sequence of at least three consecutive data points, similar to another OSA study [38]. Here, sequences of greater than three beats of either progressive increases or decreases in systolic BP and RR that were well-correlated (R ≥ 0.7) were identified. The mean slope of the regression line between these parallel sequences was calculated and used to represent the BRS, in units of ms/mmHg [18,51]. In one study, the optimal shortest recording time for BRS estimation in an orthostatic position was calculated to be 3.74 ± 0.07 min, with accuracy decreasing with shorter recording times [52]. For longer durations, the R-value threshold for well-correlated sequences was greater than 0.7 for a duration of 5 min [53,54].

### Analysis–measures:

**Fig. 3.**
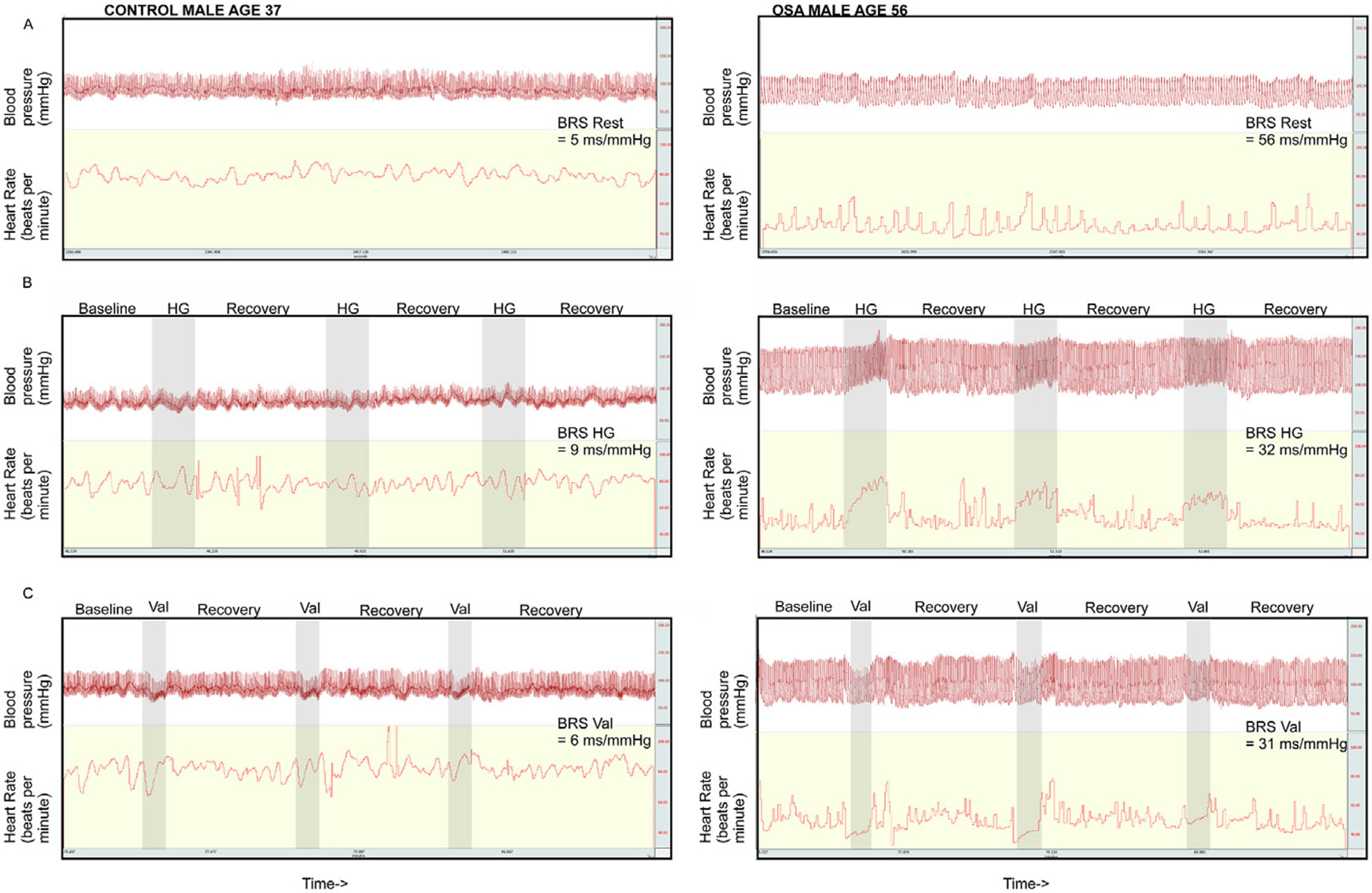
Sample traces in control and OSA participants illustrating baroreflex sensitivity (BRS) in different task periods. Examples illustrating blood pressure (BP) and heart rate (HR) patterns associated with a range of BRS values during rest (top row), handgrip (HG; middle row) and Valsalva (Val; bottom row), in control (left column) and untreated OSA (right column) participants. The x- and y-axes were similarly scaled in all graphs. Shaded areas show when a task (handgrip or Valsalva) was performed.

BRS was determined for rest, handgrip and Valsalva protocols. The regressions for ascending and descending sequences were averaged to obtain a measure of BRS during each of the the 5 min rest and two 7 min challenge periods. Fig. 3 demonstrates the average of ascending and descending sequence BRS for the periods of rest, handgrip, and Valsalva in healthy and OSA participants.

BPV was calculated from the continuous BP signal [2]. In brief, we calculated the standard deviation of beat-to-beat mean arterial BP over each of the three protocols (rest, handgrip and Valsalva), resulting in three BPV measures for each subject.

### Statistics:

We calculated descriptive statistics for BPV and BRS in the three groups (unOSA, cpOSA and CON) for the three tasks (rest, handgrip, Valsalva). Although we had three groups, our interest was in two-way comparisons, so we tested for differences in means between each pair of groups. We performed group comparisons with independent two-samples t-tests, and BPV was correlated with BRS using Pearson R correlation. We used a statistical threshold of *p* = 0.05 significance. Although the study was not powered to assess within-sex effects, we presented sex-specific findings to allow consideration of female and male effect sizes.

## 3. Results

**Fig. 4.**
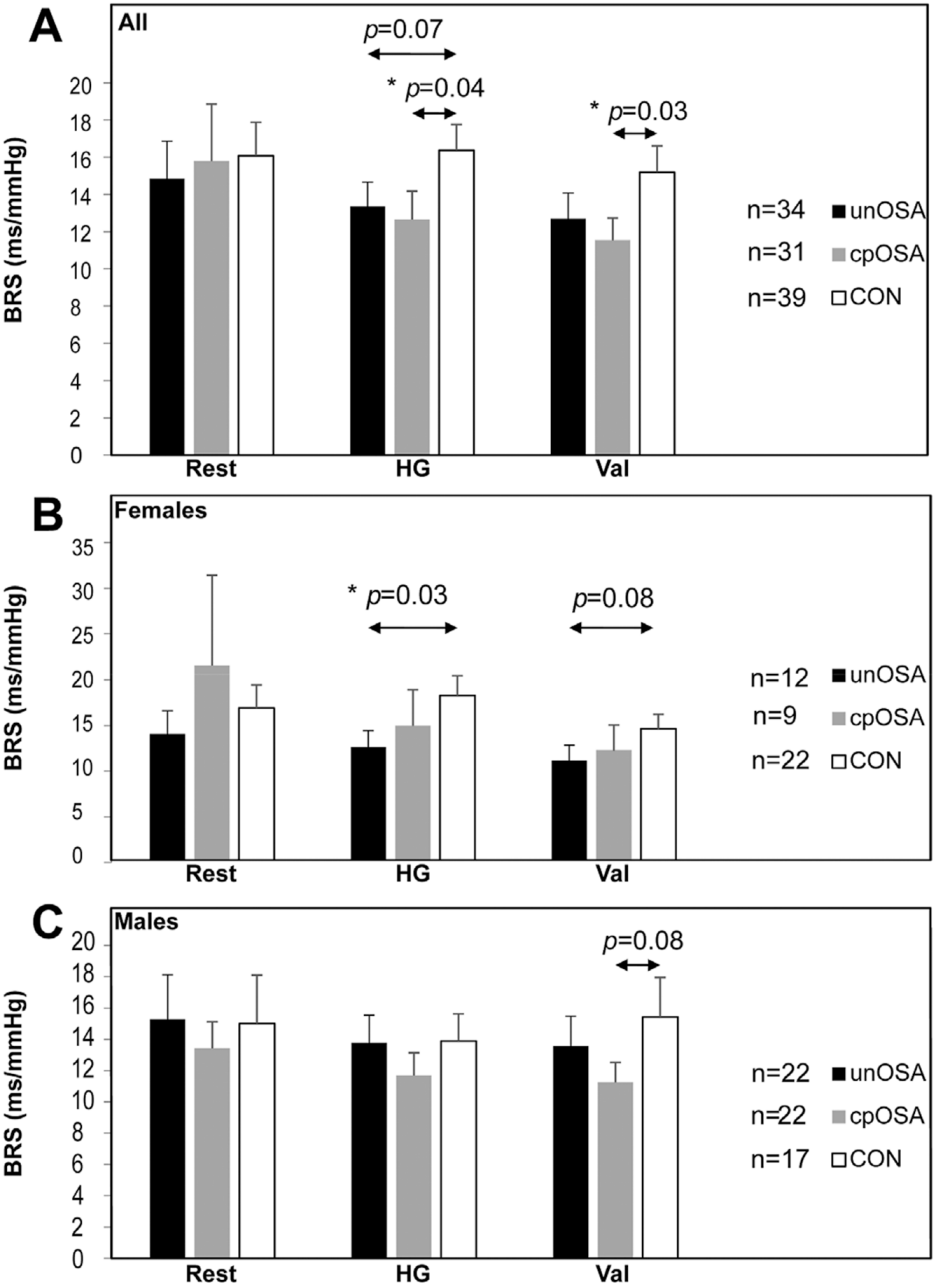
BRS changes during tasks. BRS mean ± SEM values during rest, handgrip (HG) and Valsalva (Val) are shown in untreated OSA (unOSA), CPAP treated OSA (cpOSA), and control (CON) in combined (A), female (B), and male (C) groups. *p*–values from independent two sample t-tests are shown (*≤0.05).

Resting BRS was not significantly different between groups (Fig. 4 and Table 2). Group BRS differences emerged during handgrip, with significantly lower values in both OSA groups compared with the control participants (unOSA mean±SEM ms/mmHg: 13.3±1.3; cpOSA: 12.7±1.5; CON: 16.4±1.4; Fig. 4 and Table 2). Similarly, OSA BRS was lower during Valsalva (unOSA mean±SEM ms/mmHg: 12.7±1.4; cpOSA: 11.5±1.2; CON: 15.1±1.4). BRS was negatively correlated with BPV in OSA during Valsalva for unOSA and during handgrip for cpOSA, both at R = −0.4 (*p* < 0.05; Table 3). BRS was negatively correlated with OSA severity (levels: none, mild, moderate, severe) at R = −0.2 (*p* < 0.05; Table 3).

**Fig. 5.**
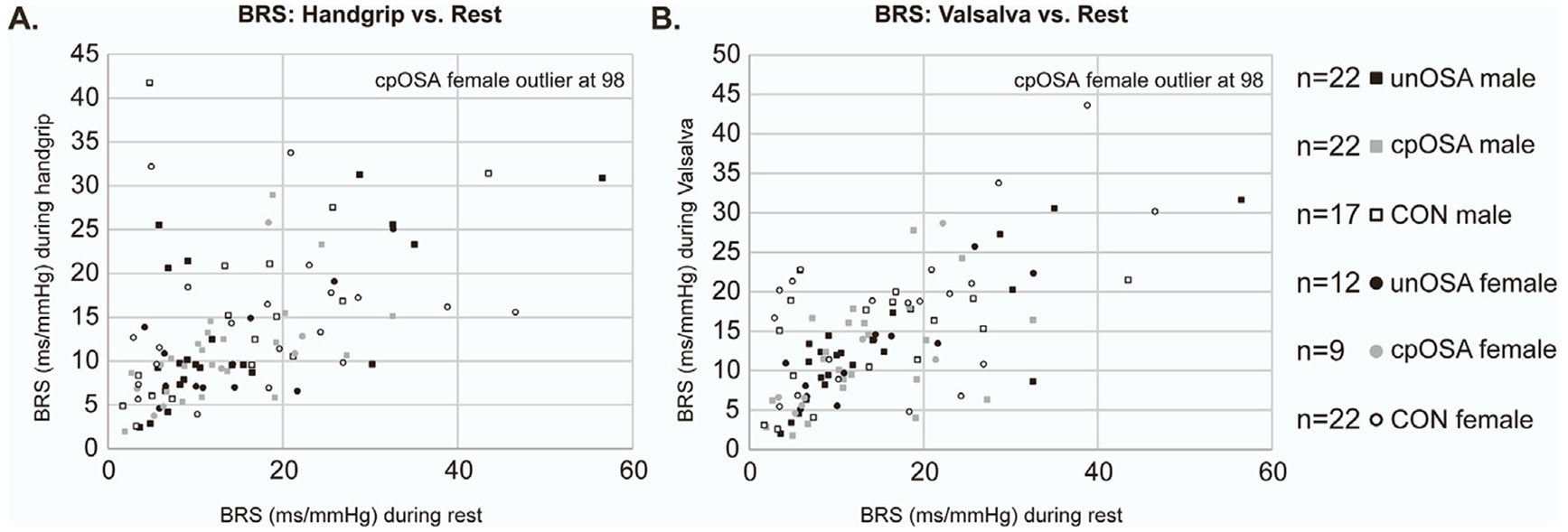
BRS correlations between rest and task. Scatterplots of BRS (ms/mmHg) of (A) Valsalva vs rest, and (B) handgrip vs rest in untreated OSA (unOSA), CPAP treated OSA (cpOSA), and control (CON).

**Fig. 6.**
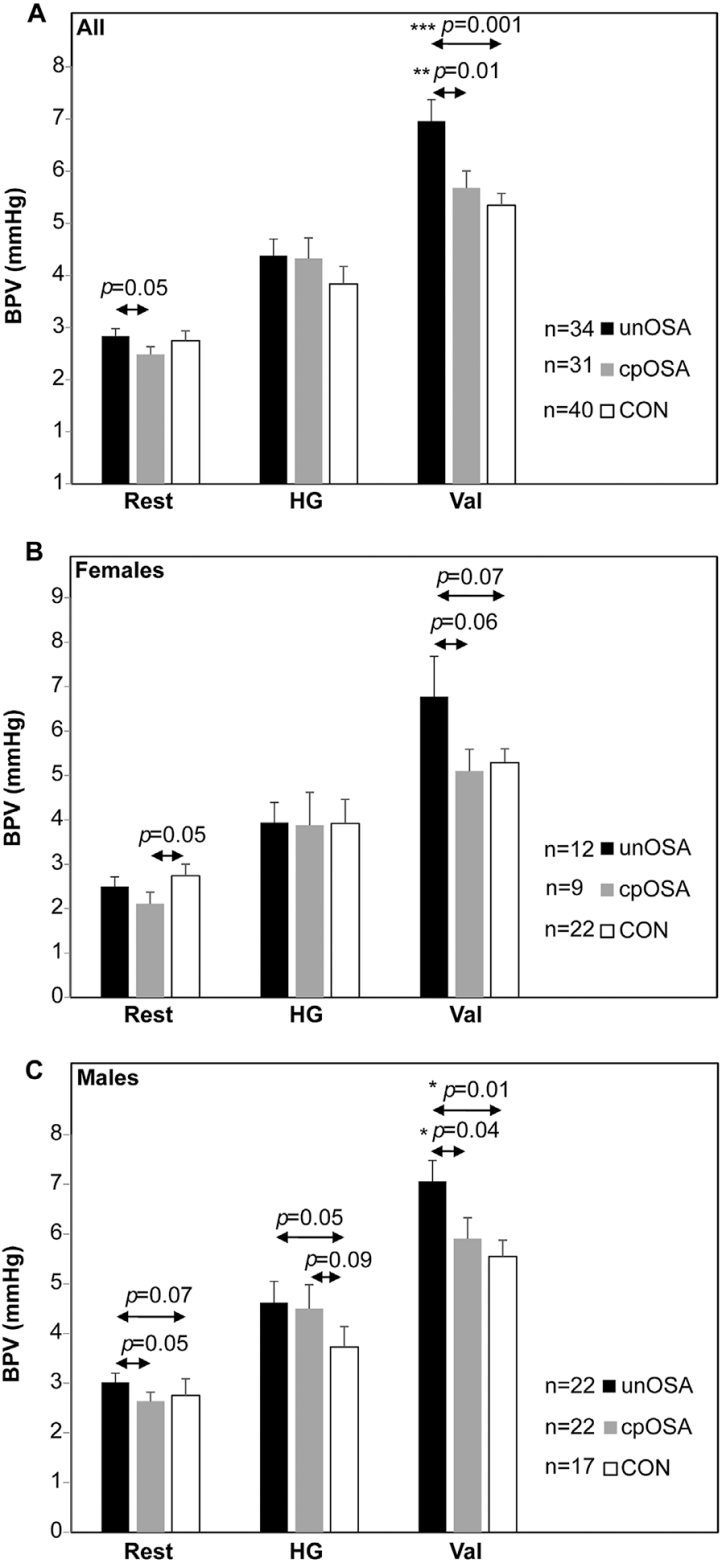
BPV changes during tasks. BPV mean ± SEM values during rest, handgrip (HG) and Valsalva (Val) are shown in untreated OSA (unOSA), CPAP treated OSA (cpOSA) and control (CON) in the combined sample (A), females (B) and males (C). *p*–values from independent two sample t-tests are shown (* <0.05, ** <0.01, *** <0.001).

**Fig. 7.**
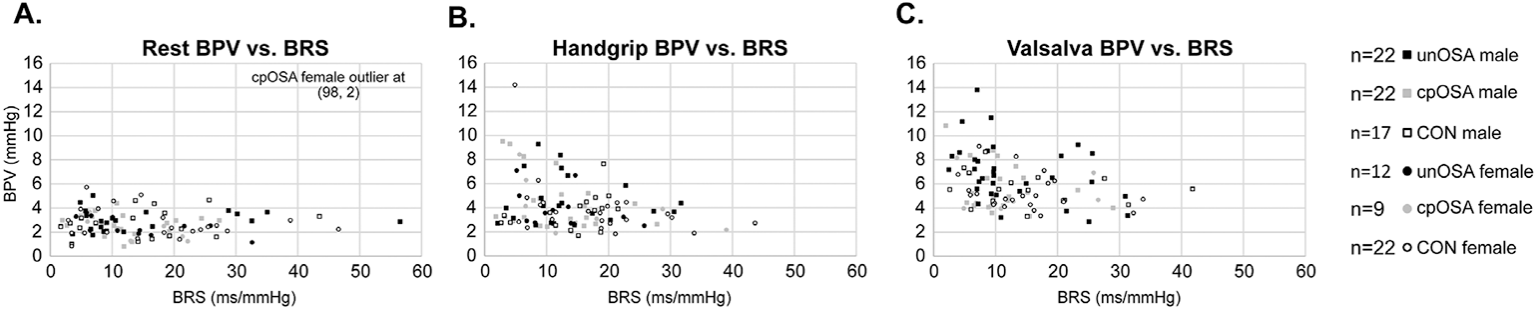
BRS and BPV correlations. Scatterplots of BPV (mmHg) vs BRS (ms/mmHg) in untreated OSA (unOSA), CPAP treated OSA (cpOSA), and control (CON) during (A) rest, (B) handgrip (HG) and (C) Valsalva (Val).

Additional findings illustrate relationships of BRS with other variables. Scatterplots of pressor-induced BRS with BRS at rest illustrate associations across groups (Fig. 5). BPV (standard deviation of beat-to-beat BP) was similar across groups during rest and handgrip, but was significantly lower in CON during Valsalva (Table 2, Fig. 6). BRS was negatively correlated with BPV when assessed over all participants; however, in group-specific analyses, this relationship was only significant in unOSA during Valsalva (R = −0.4, *p* = 0.02) and in cpOSA during handgrip (also R = −0.4, *p* = 0.02) (Table 3, Fig. 7). BRS for handgrip and Valsalva correlated negatively with OSA severity when assessed in all participants (n = 104, levels: none, mild, moderate, severe) at R = −0.2, *p* = 0.04 (Table 2).

## 4. Discussion

We found that BRS is depressed in OSA relative to healthy participants irrespective of CPAP usage during the autonomic challenges of handgrip and Valsalva, but not at rest. The lack of depressed BRS during rest in OSA vs healthy is consistent with other studies [29]. Additionally, the range of disease severity in the present data may also contribute to only modest differences, since depressed BRS is strongly associated with severe OSA [55], but to a lesser degree with mild or moderate OSA [6]. In our sample, there were more-mild and moderate rather than severe cases in the untreated OSA group compared to the CPAP group, so this difference in severity may partially explain the more depressed BRS in CPAP vs untreated OSA. We did find BRS correlated negatively with OSA severity, consistent with previous studies [6,25,37]. These influences notwithstanding, in the present sample we did not find evidence to support the possibility that BRS is improved with CPAP.

Existing literature suggests that with increased OSA severity, higher sympathetic activity and decreased BRS are common even during wakefulness, and that CPAP treatment improves the baroreflex during sleep [1,6,25]. The findings here suggest that altered BRS during wakefulness may not be addressed by CPAP usage, a finding supported by some previous literature [56], although the cross-sectional nature of the present study limits this interpretation. CPAP in the present participants likely has some effect on autonomic function, since BPV in that group is lower than in untreated OSA during rest.

The reduced BRS in OSA was associated with patients taking longer to recover BP following handgrip and Valsalva as reflected in the negative correlation of BRS with BPV. However, the relationship between BRS and BPV was variable; BPV was higher untreated but not CPAP OSA or healthy controls. BP is typically higher in OSA [57], and bradycardia [58] and lowered HRV [59] are common. The altered resting HRV could be another reflection of the reduced BRS has shown during pressor challenges. These OSA effects may reflect a weakened response, rather than a missing or abnormal one, since we previously found that the insular functional organization during handgrip had similar pattern time-courses in OSA and CON groups, with only magnitude differences in the neural responses [46]. Overall, it appears that although OSA participants can respond to rapid changes in BP, the baroreflex function is less effective than in healthy people, leading to poor BP and HR control.

Depressed BRS may negatively affect cardiovascular health in OSA [60], which would be of particular concern if BRS does not resolve with CPAP, at least during wakefulness [61]. Although there are reports of restoration of BRS and autonomic functioning after CPAP [17], those reports should be considered in the context of resting levels versus particular autonomic challenges that modify BRS responsiveness. In normotensive OSA patients, sympathetic nervous system activity, based on norepinephrine excretion, is continuously increased and is not affected by short-term CPAP treatment [61]. Nevertheless, treatment presumably does help mitigate pathological physiology, as CPAP reduces sympathetic tone as measured by MSNA [15] and during sleep CPAP improves baroreflex function [24]. Over time, such benefits would presumably reduce the incidence or severity of cardiovascular disease.

The neural changes induced by OSA likely contribute at least in part to the processes underlying the baroreflex findings. OSA damages the insula, especially the right insula [9,10,39], and such damage is associated with depressed BRS and poor cardiac regulation generally [62-67]. The finding suggesting that CPAP may not correct that loss likely derives from that region-specific injury, and may imply long-term deficits; although glial cells may be replaced, neurons in the insula are not [10]. Intermittent hypoxic injury in OSA appears in the deep cerebellar nuclei, cerebellar Purkinje cells, and the parabrachial pons; the same brain regions are known to control HRV [15,68-70]. One role of the deep cerebellar and parabrachial pontine nuclei is to integrate incoming afferent information from the lungs and other parts of the respiratory system with cardiovascular output processes [71], and with injury, that cardiovascular control is compromised in OSA [72]. BP control has several hierarchal levels; at rest, low level, reflexive mechanisms are prominent and may be less affected by OSA injury (because they operate in areas less affected by hypoxic injury). However, as in major non-rest conditions (challenges such as breathing stress and rapid BP changes), additional control processes are brought into action by cortical regions, including the insula, which are largely compromised in OSA. Determining the role of injured structures in contributing to BRS should be a goal of future studies.

### Limitations:

Any medication that alters BP could impact the overall findings of our study; we only excluded medicines known to have a major cardiovascular impact. Both OSA groups were older than the control groups, which presumably would have enhanced rather than obscured any disease-related differences. The CPAP adherence was self-reported, and it is possible the treated group included a range of hours/night and nights/week of usage. The similar ages of treated vs untreated groups mean that CPAP vs untreated comparisons were likely not unduly affected by age. As expected, the OSA group showed more signs of cardiovascular disease, with four participants in each of the OSA groups reporting hypertension vs two in the control group. The cross-sectional nature of the study limits any causal inferences. The mixture of mild, moderate and severe OSA patients likely diluted the findings that might be apparent in a severe group of patients, such as spontaneous BRS during rest which we would expect to be affected in that subgroup. Finally, the study was not powered to identify for sex differences, so the sex-specific findings should be considered trends that remain to be confirmed.

### Conclusions:

BRS was not reduced during rest in OSA, but was depressed during handgrip and Valsalva challenges. Higher BPV correlated with the decreased BRS during the autonomic challenges, suggesting impaired acute BP regulation in OSA. High OSA severity was a predictor of depressed BRS, consistent with existing literature [1,6]. The present findings do not provide evidence that CPAP addresses the depressed BRS in OSA in the awake state, despite previous indications that CPAP treatment alters the BRS during sleep, especially in severe OSA [24,25]. These findings raise the question of which mechanisms of cardiac regulation are resolved with CPAP, and which are based on other pathology such as brain injury that may need alternative treatments.

## Figures and Tables

**Table 1:** Participant details. Participant characteristics by group and sex. Measures are presented as mean±stdev [range]. BMI: body mass index; CON: control; cpOSA: CPAP-treated OSA; unOSA: untreated OSA; REI: respiratory event index.

	unOSA			cpOSA			CON		
	All (N= 34)	Males (N= 22)	Females (N= 12)	All (N= 31)	Males (N= 22)	Females (N= 9)	All (N= 39)	Males (N=17)	Females (N= 22)
Age (years)†	50.6±14.2 [25–77]	47.4±13.7 [25–77]	56.5±13.6 [34–77]	49.6±14.5 [27–73]	46.0±14.2 [27–71]	57.9±12.2 [31–73]	42.1±15.0 [21–67]	42.1±16.5 [23–67]	42.2±14.4 [21–66]
BMI (kg/m^2^)	32.8±8.0 [21.9–54.3]	32.8±7.3 [22.2–47.0]	31.7±7.40 [21.9–54.3]	29.4±5.5 [19.6–42]	28.4±4.4 [19.6–38.3]	29.4±5.5 [22.6–42]	26.0±4.3 [19.6–37.6]	27.2±3.6 [21.8–33.2]	25.2±4.7[19.6–37.6]
Sleep parameters									
REI (events/hour)	21.1±15.3 [6.0–67.4]	19.1±10.7 [6.0–42.0]	24.7±21.3 [6.9–67.4]	22.5±13.9 [5.0–58]	21.4±13.9 [5.0–58.0]	25.5±14.3 [6.0–45.9]			
Mean oxygen saturation (%)	94.8±1.5 [91–96.5]	94.2±2.3 [90.0–99.9]	94.8±1.5 [92.0–96.5]	94.0±2.2 [91.0–96.5]	94.8±1.6 [91.0–96.4]	93.8±2.2 [92.0–96.5]			
Minimum oxygen saturation (%)	83.6±5.8 [68.8–92.0]	83.6±5.5 [68.8–92.0]	82.4±7.1 [67.0–91.4]	82.7±9.0 [68.8–92.0]	82.4±7.1 [68.8–92.0]	83.4±13.4 [70.9–92.0]			

† *p*<0.01 differences in age between CON and both OSA groups (unOSA and cpOSA). No significant difference was present between unOSA and cpOSA.

**Table 2:** Physiological measures. The main outcome variable of BRS (ms/mmHg) and secondary outcome variable BPV (mmHg) during rest, handgrip (HG) and Valsalva (Val) for unOSA, cpOSA and CON are presented as mean±stdev [range]. Significance values for two-group comparisons using independent two-sample t tests are shown, with *p* < 0.05, *p* < 0.01 and *p* < 0.001 indicated by *, ** and ***, respectively. BPV: blood pressure variability; BRS: baroreflex sensitivity; CON: control; cpOSA: CPAP-treated OSA; unOSA: untreated OSA.

All	unOSA (N=34:	cpOSA (N=31:	CON (N=39)	†	‡	††
	15 mild,	11 mild,				
	11 moderate,	8 moderate,				
	8 severe)	12 severe)				
BRS at rest (ms/mmHg)	14.8±11.8 [3.6–56.5]	15.8±17.0 [1.9–98.3]	16.1±11.3 [1.7–46.5]	0.34	0.47	0.41
	Mild: 12.8±8.7	Mild: 21.5±27.2				
	Moderate: 16.9±15.5	Moderate: 13.3±8.0				
	Severe: 16.0±11.8	Severe: 12.2±4.1				
BRS at HG (ms/mmHg)	13.3±7.6 [3.5–31.7]	12.7±8.4 [1.9–39.0]	16.4±8.7 [1.6–43.6]	0.07	*0.04	0.35
	Mild: 13.6±7.1	Mild: 14.6±10.0				
	Moderate: 13.6±7.5	Moderate: 9.5±5.1				
	Severe: 12.5±9.3	Severe: 12.9±7.9				
BRS at Val (ms/mmHg)	12.7±8.0 [2.4–31.3]	11.5±6.6 [1.9–29.0]	15.1±8.9 [2.6–41.8]	0.11	*0.03	0.27
	Mild: 13.4±7.6	Mild: 12.3±7.1				
	Moderate: 13.5±9.2	Moderate: 12.4±6.6				
	Severe: 10.3±7.9	Severe: 10.3±7.3				
BPV at rest (mmHg)	2.8±0.8 [1.2–5.1]	2.5±0.8 [0.8–4.4]	2.8±0.8 [0.9–5.7]	0.36	0.14	*0.05
	Mild: 2.9±0.9	Mild: 2.5±0.8				
	Moderate: 2.8±0.6	Moderate: 2.7±0.5				
	Severe: 2.6±1.1	Severe: 2.3±1.1				
BPV at HG (mmHg)	4.4±1.9 [2.5–9.3]	4.3±2.2 [1.9–9.5]	3.8±2.4 [1.7–14.2]	0.12	0.17	0.46
	Mild: 4.2±1.9	Mild: 4.9±2.3				
	Moderate: 4.9±1.9	Moderate: 5.0±2.6				
	Severe: 3.8±1.7	Severe: 3.3±1.7				
BPV at Val (mmHg)	7.0±2.4 [2.9–13.8]	5.7±1.8 [3.3–10.8]	5.3±1.4 [3.3–9.1]	***0.0006	0.2	**0.008
	Mild: 7.2±3.2	Mild: 5.3±1.7				
	Moderate: 6.8±1.5	Moderate: 6.5±2.1				
	Severe: 6.8±1.7	Severe: 5.4±1.6				
Males	unOSA (N=22)	cpOSA (N=22)	CON (N=17)	†	‡	††
BRS at rest (ms/mmHg)	15.3±13.7 [3.6–56.5]	13.4±8.0 [1.9–32.5]	15.0±10.7 [1.6–43.5]	0.47	0.31	0.29
BRS at HG (ms/mmHg)	13.8±8.3 [2.1–31.7]	11.7±6.7 [1.8–27.8]	13.8±6.1 [2.6–21.5]	0.48	0.15	0.18
BRS at Val (ms/mmHg)	13.1±7.6 [2.1–31.7]	13.7±9.0 [1.8–39.0]	18.3±11.9 [3.1–62.9]	0.28	0.08	0.16
BPV at rest (mmHg)	3.1±0.8 [1.8–5.1]	2.6±0.8 [0.8–4.4]	2.7±1.1 [1.0–4.7]	0.22	0.36	0.08
BPV at HG (mmHg)	4.6±2.0 [2.6–9.3]	4.5±2.2 [2.4–9.5]	3.7±1.4 [1.7–7.7]	0.06	0.10	0.43
BPV at Val (mmHg)	7.1±1.9 [3.4–11.5]	5.9±1.9 [3.3–10.8]	5.5±1.4 [3.3–8.7]	**0.003	0.25	*0.03
Females	unOSA (N=12)	cpOSA (N=9)	CON (N=22)	†	‡	††
BRS at rest (ms/mmHg)	14.1±8.8 [4.1–32.6]	21.5±29.6 [3.3–98.3]	16.9±11.8 [2.8–46.5]	0.22	0.33	0.24
BRS at HG (ms/mmHg)	12.6±6.4 [5.2–25.7]	14.9±11.8 [4.6–38.9]	18.2±10.0 [4.9–43.6]	*0.03	0.23	0.30
BRS at Val (ms/mmHg)	11.1±6.1 [4.6–25.1]	12.3±12.2 [3.8–26.5]	14.6±7.7 [3.9–33.8]	0.08	0.24	0.36
BPV at rest (mmHg)	2.5±0.7 [1.2–3.6]	2.1±0.8 [1.3–3.7]	2.7±1.2 [0.9–5.7]	0.24	*0.05	0.14
BPV at HG (mmHg)	3.9±1.6 [2.5–7.0]	3.9±2.2 [1.9–8.4]	3.9±2.5 [1.9–14.2]	0.49	0.48	0.47
BPV at Val (mmHg)	6.7±3.1 [2.8–13.8]	5.1±1.4 [3.9–8.1]	5.3±1.5 [3.4–9.2]	0.07	0.37	0.06

† *p*-value unOSA vs. CON

‡ *p*-value cpOSA vs. CON

†† *p*-value unOSA vs. CPAP.

**Table 3:** Correlations between BRS and BPV. The Person R correlation values for BRS (ms/mmHg) and BPV (mmHg) for unOSA, cpOSA and CON are presented during rest, handgrip (HG) and Valsalva (Val). * indicates *p* < 0.05 as per Fisher Z-transformation. BPV: blood pressure variability; BRS: baroreflex sensitivity; CON: control; cpOSA: CPAP-treated OSA; unOSA: untreated OSA.

Correlation of BRS and BPV	All			Male			Female		
Tasks	R, unOSA N = 34	R, cpOSA N = 31	R, CON N = 39	R, unOSA N = 22	R, cpOSA N = 22	R, CON N = 17	R, unOSA N = 12	R, cpOSA N = 9	R, CON N = 22
Rest	−0.03	−0.21	−0.04	0.14	−0.06	0.21	−0.67*	−0.29	−0.20
HG	−0.14	−0.41*	−0.23	−0.08	−0.41	0.37	−0.38	−0.43	−0.41
Val	−0.37*	−0.24	−0.26	−0.36	−0.38	−0.21	−0.53	0.09	−0.30

## Abbreviations

AASM: American Academy of Sleep Medicine
ANS: autonomic nervous system
BMI: body mass index
BP: blood pressure
BPV: mean arterial blood pressure variability
BRS: Baroreflex sensitivity
CON: control
CPAP: continuous positive airway pressure
cpOSA: CPAP-treated OSA
CVD: cardiovascular disorders
HR: heart rate
HRV: heart rate variability
HSAT: home sleep apnea testing
MBP: mean arterial blood pressure
MSNA: muscle sympathetic nerve activity
OSA: obstructive sleep apnea
REI: respiratory event index
RR–I (t): RR–interval or heart period
SBP: systolic blood pressure
unOSA: untreated OSA

## References

[1] Lombardi C, Pengo MF, Parati G. Obstructive sleep apnea syndrome and autonomic dysfunction. Auton Neurosci 2019;221:102563. 31445406 10.1016/j.autneu.2019.102563

[2] Pal A, Martinez F, Aguila AP, Akey MA, Chatterjee R, Conserman MGE, Beat-to-beat blood pressure variability in patients with obstructive sleep apnea. J Clin Sleep Med 2021;17:381–92. 33089774 10.5664/jcsm.8866 PMC7927319

[3] Mansukhani MP, Kara T, Caples SM, Somers VK. Chemoreflexes, sleep apnea, and sympathetic dysregulation. Curr Hypertens Rep 2014;16:476. 25097113 10.1007/s11906-014-0476-2 PMC4249628

[4] Patel AR, Patel AR, Singh S, Singh S, Khawaja I. The association of obstructive sleep apnea and hypertension. Cureus 2019;11. e4858–e. 31410341 10.7759/cureus.4858 PMC6684296

[5] Dopp JM, Reichmuth KJ, Morgan BJ. Obstructive sleep apnea and hypertension: mechanisms, evaluation, and management. Curr Hypertens Rep 2007;9: 529–34. 18367017 10.1007/s11906-007-0095-2

[6] Blomster H, Laitinen TP, Hartikainen JE, Laitinen TM, Vanninen E, Gylling H, Mild obstructive sleep apnea does not modulate baroreflex sensitivity in adult patients. Nat Sci Sleep 2015;7:73–80. 26203292 10.2147/NSS.S82443 PMC4487157

[7] Monahan KD, Leuenberger UA, Ray CA. Effect of repetitive hypoxic apnoeas on baroreflex function in humans. J Physiol 2006;574:605–13. 16709638 10.1113/jphysiol.2006.108977 PMC1817765

[8] Kumar R, Birrer BV, Macey PM, Woo MA, Gupta RK, Yan-Go FL, Reduced mammillary body volume in patients with obstructive sleep apnea. Neurosci Lett 2008;438:330–4. 18486338 10.1016/j.neulet.2008.04.071

[9] Macey PM, Kumar R, Woo MA, Valladares EM, Yan-Go FL, Harper RM. Brain structural changes in obstructive sleep apnea. Sleep 2008;31:967–77. 18652092 PMC2491498

[10] Park B, Palomares JA, Woo MA, Kang DW, Macey PM, Yan-Go FL, Aberrant insular functional network integrity in patients with obstructive sleep apnea. Sleep 2016;39:989–1000. 26943471 10.5665/sleep.5738 PMC4835320

[11] Dampney RAL. Resetting of the baroreflex control of sympathetic vasomotor activity during natural behaviors: description and conceptual model of central mechanisms. Front Neurosci 2017;11:461. 28860965 10.3389/fnins.2017.00461 PMC5559464

[12] Henderson LA, Macefield VG. Obstructive sleep apnoea and hypertension: the role of the central nervous system. Curr Hypertens Rep 2016;18:59. 27278369 10.1007/s11906-016-0665-2

[13] Lundblad LC, Fatouleh RH, Hammam E, McKenzie DK, Macefield VG, Henderson LA. Brainstem changes associated with increased muscle sympathetic drive in obstructive sleep apnoea. Neuroimage 2014;103:258–66. 25255048 10.1016/j.neuroimage.2014.09.031

[14] Macey PM, Sarma MK, Prasad JP, Ogren JA, Aysola R, Harper RM, Obstructive sleep apnea is associated with altered midbrain chemical concentrations. Neuroscience 2017. 10.1016/j.neuroscience.2017.09.001 PMC5983363 28893651

[15] Fatouleh RH, Hammam E, Lundblad LC, Macey PM, McKenzie DK, Henderson LA, Functional and structural changes in the brain associated with the increase in muscle sympathetic nerve activity in obstructive sleep apnoea. Neuroimage Clin 2014;6:275–83. 25379440 10.1016/j.nicl.2014.08.021 PMC4215471

[16] Fatouleh RH, Lundblad LC, Macey PM, McKenzie DK, Henderson LA, Macefield VG. Reversal of functional changes in the brain associated with obstructive sleep apnoea following 6 months of CPAP. Neuroimage Clin 2015;7:799–806. 26082888 10.1016/j.nicl.2015.02.010 PMC4459270

[17] Lundblad LC, Fatouleh RH, McKenzie DK, Macefield VG, Henderson LA. Brain stem activity changes associated with restored sympathetic drive following CPAP treatment in OSA subjects: a longitudinal investigation. J Neurophysiol 2015;114:893–901. 25995345 10.1152/jn.00092.2015 PMC4533106

[18] Iellamo F, Legramante JM, Raimondi G, Peruzzi G. Baroreflex control of sinus node during dynamic exercise in humans: effects of central command and muscle reflexes. Am J Physiol Heart Circ Physiol 1997;272:H1157–64. 10.1152/ajpheart.1997.272.3.H1157 9087588

[19] Lundblad LC, Fatouleh RH, Hammam E, McKenzie DK, Macefield VG, Henderson LA. Brainstem changes associated with increased muscle sympathetic drive in obstructive sleep apnoea. Neuroimage 2014;103:258–66. 25255048 10.1016/j.neuroimage.2014.09.031

[20] Macefield VG, Henderson LA. Identifying increases in activity of the human RVLM through MSNA-coupled fMRI. Front Neurosci 2020;13. 10.3389/fnins.2019.01369 PMC6985468 32038124

[21] Parati G. Arterial baroreflex control of heart rate: determining factors and methods to assess its spontaneous modulation. J Physiol 2005;565:706–7. 15878943 10.1113/jphysiol.2005.086827 PMC1464559

[22] Kohli P, Balachandran JS, Malhotra A. Obstructive sleep apnea and the risk for cardiovascular disease. Curr Atherosclerosis Rep 2011;13:138–46. 10.1007/s11883-011-0161-8 PMC4332589 21253882

[23] Sabino-Carvalho JL, Falquetto B, Takakura AC, Vianna LC. Baroreflex dysfunction in Parkinson's disease: integration of central and peripheral mechanisms. J Neurophysiol 2021;125:1425–39. 33625931 10.1152/jn.00548.2020

[24] Bonsignore MR, Parati G, Insalaco G, Marrone O, Castiglioni P, Romano S, Continuous positive airway pressure treatment improves baroreflex control of heart rate during sleep in severe obstructive sleep apnea syndrome. Am J Respir Crit Care Med 2002;166:279–86. 12153958 10.1164/rccm.2107117

[25] Bonsignore MR, Parati G, Insalaco G, Castiglioni P, Marrone O, Romano S, Baroreflex control of heart rate during sleep in severe obstructive sleep apnoea: effects of acute CPAP. Eur Respir J 2006;27:128. 16387945 10.1183/09031936.06.00042904

[26] Carlson JT, Hedner J, Elam M, Ejnell H, Sellgren J, Wallin BG. Augmented resting sympathetic activity in awake patients with obstructive sleep apnea. Chest 1993;103:1763–8. 8404098 10.1378/chest.103.6.1763

[27] Narkiewicz K, Somers VK. Sympathetic nerve activity in obstructive sleep apnoea. Acta Physiol Scand 2003;177:385–90. 12609010 10.1046/j.1365-201X.2003.01091.x

[28] Carlson JT, Hedner JA, Sellgren J, Elam M, Wallin BG. Depressed baroreflex sensitivity in patients with obstructive sleep apnea. Am J Respir Crit Care Med 1996;154:1490–6. 8912770 10.1164/ajrccm.154.5.8912770

[29] Narkiewicz K, Pesek CA, Kato M, Phillips BG, Davison DE, Somers VK. Baroreflex control of sympathetic nerve activity and heart rate in obstructive sleep apnea. Hypertension 1998;32:1039–43. 9856970 10.1161/01.hyp.32.6.1039

[30] Parati G, Di Rienzo M, Bonsignore MR, Insalaco G, Marrone O, Castiglioni P, Autonomic cardiac regulation in obstructive sleep apnea syndrome: evidence from spontaneous baroreflex analysis during sleep. J Hypertens 1997;15:1621–6. 9488213 10.1097/00004872-199715120-00063

[31] Dewan NA, Nieto FJ, Somers VK. Intermittent hypoxemia and OSA: implications for comorbidities. Chest 2015;147:266–74. 25560865 10.1378/chest.14-0500 PMC4285080

[32] Narkiewicz K, Kato M, Phillips BG, Pesek CA, Davison DE, Somers VK. Nocturnal continuous positive airway pressure decreases daytime sympathetic traffic in obstructive sleep apnea. Circulation 1999;100:2332–5. 10587337 10.1161/01.cir.100.23.2332

[33] Henderson LA, Fatouleh RH, Lundblad LC, McKenzie DK, Macefield VG. Effects of 12 Months continuous positive airway pressure on sympathetic activity related brainstem function and structure in obstructive sleep apnea. Front Neurosci 2016;10:90. 27013952 10.3389/fnins.2016.00090 PMC4785184

[34] Swenne CA. Baroreflex sensitivity: mechanisms and measurement. Neth Heart J : Monthly J Netherlands Soc Cardiol Netherlands Heart Foundation 2013;21: 58–60. 10.1007/s12471-012-0346-y PMC3547418 23179611

[35] Martin CE, Shaver JA, Leon DF, Thompson ME, Reddy PS, Leonard JJ. Autonomic mechanisms in hemodynamic responses to isometric exercise. J Clin Invest 1974;54:104–15. 4600046 10.1172/JCI107731 PMC301529

[36] Idiaquez J, Idiaquez JF, Iturriaga R. Cardiovascular responses to isometric handgrip exercise in young patients with recurrent vasovagal syncope. Auton Neurosci 2018;212:23–7. 29778242 10.1016/j.autneu.2018.04.001

[37] Chen H-L, Huang C-C, Lin H-C, Lu C-H, Chen P-C, Chou K-H, White matter alteration and autonomic impairment in obstructive sleep apnea. J Clin Sleep Med 2020;16:293–302. 31992402 10.5664/jcsm.8186 PMC7053037

[38] Araújo CEL, Ferreira-Silva R, Gara EM, Goya TT, Guerra RS, Matheus L, Effects of exercise training on autonomic modulation and mood symptoms in patients with obstructive sleep apnea. Braz J Med Biol Res 2021;54. e10543–e. 33729391 10.1590/1414-431X202010543 PMC7959152

[39] Pal A, Ogren JA, Aguila AP, Aysola R, Kumar R, Henderson LA, Functional organization of the insula in men and women with obstructive sleep apnea during Valsalva. Sleep 2020. 10.1093/sleep/zsaa124 PMC7819841 32592491

[40] Macey PM, Kumar R, Woo MA, Yan-Go FL, Harper RM. Heart rate responses to autonomic challenges in obstructive sleep apnea. PLoS One 2013;8:e76631. 24194842 10.1371/journal.pone.0076631 PMC3806804

[41] Tamuli D, Kaur M, Boligarla A, Jaryal AK, Srivastava AK, Deepak KK. Depressed baroreflex sensitivity from spontaneous oscillations of heart rate and blood pressure in SCA1 and SCA2. Acta Neurol Scand 2019;140:350–8. 31343735 10.1111/ane.13151

[42] Narkiewicz K, Somers VK. Cardiovascular variability characteristics in obstructive sleep apnea. Auton Neurosci 2001;90:89–94. 11485297 10.1016/S1566-0702(01)00272-7

[43] Marrone O, Bonsignore MR. Blood-pressure variability in patients with obstructive sleep apnea: current perspectives. Nat Sci Sleep 2018;10:229–42. 30174467 10.2147/NSS.S148543 PMC6109653

[44] Man T, Tegegne BS, van Roon AM, Rosmalen JGM, Nolte IM, Snieder H, Spontaneous baroreflex sensitivity and its association with age, sex, obesity indices and hypertension: a population study. Am J Hypertens 2021;34: 1276–83. 34329370 10.1093/ajh/hpab122

[45] Macey PM, Rieken NS, Ogren JA, Macey KE, Kumar R, Harper RM. Sex differences in insular cortex gyri responses to a brief static handgrip challenge. Biol Sex Differ 2017;8:13. 28435658 10.1186/s13293-017-0135-9 PMC5397762

[46] Pal A, Ogren JA, Aysola RS, Kumar R, Henderson LA, Harper RM, Insular functional organization during handgrip in females and males with obstructive sleep apnea. PLoS One 2021;16:e0246368. 33600443 10.1371/journal.pone.0246368 PMC7891756

[47] Ayappa I, Norman RG, Seelall V, Rapoport DM. Validation of a self-applied unattended monitor for sleep disordered breathing. J Clin Sleep Med 2008;4:26–37. 18350959 PMC2276822

[48] Berry RB, Purdy S, Kantner G, Blase A, Javed F, Benjafield A, 0463 validation of a home sleep apnea testing device for the diagnosis of sleep disordered breathing based on AASM 2012 guidelines. Sleep 2019;42. A186–A.

[49] Fortin J, Marte W, Grullenberger R, Hacker A, Habenbacher W, Heller A, Continuous non-invasive blood pressure monitoring using concentrically interlocking control loops. Comput Biol Med 2006;36:941–57. 16483562 10.1016/j.compbiomed.2005.04.003

[50] Westerhof BE, Gisolf J, Karemaker JM, Wesseling KH, Secher NH, van Lieshout JJ. Time course analysis of baroreflex sensitivity during postural stress. Am J Physiol Heart Circ Physiol 2006;291:H2864–74. 16861691 10.1152/ajpheart.01024.2005

[51] Pinna GD, Maestri R, La Rovere MT. Assessment of baroreflex sensitivity from spontaneous oscillations of blood pressure and heart rate: proven clinical value? Physiol Meas 2015;36:741–53. 25798657 10.1088/0967-3334/36/4/741

[52] Martínez-García P, Lerma C, Infante O. Baroreflex sensitivity estimation by the sequence method with delayed signals. Clin Auton Res 2012;22:289–97. 22875549 10.1007/s10286-012-0173-7

[53] Parati G, Di Rienzo M, Castiglioni P, Bouhaddi M, Cerutti C, Cividjian A, Assessing the sensitivity of spontaneous baroreflex control of the heart: deeper insight into complex physiology. Hypertension 2004;43:e32–4. 15051672 10.1161/01.HYP.0000126689.12940.cd

[54] Parati G, Saul JP, Castiglioni P. Assessing arterial baroreflex control of heart rate: new perspectives. J Hypertens 2004;22:1259–63. 15201539 10.1097/01.hjh.0000125469.35523.32

[55] Ryan S, Ward S, Heneghan C, McNicholas WT. Predictors of decreased spontaneous baroreflex sensitivity in obstructive sleep apnea syndrome. Chest 2007;131:1100–7. 17426215 10.1378/chest.06-2165

[56] Belozeroff V, Berry RB, Sassoon CSH, Khoo MCK. Effects of CPAP therapy on cardiovascular variability in obstructive sleep apnea: a closed-loop analysis. Am J Physiol Heart Circ Physiol 2002;282:H110–21. 11748054 10.1152/ajpheart.2002.282.1.H110

[57] Ke X, Sun Y, Yang R, Liang J, Wu S, Hu C, Association of 24 h-systolic blood pressure variability and cardiovascular disease in patients with obstructive sleep apnea. BMC Cardiovasc Disord 2017;17:287. 29212465 10.1186/s12872-017-0723-y PMC5719739

[58] Zwillich C, Devlin T, White D, Douglas N, Weil J, Martin R. Bradycardia during sleep apnea. Characteristics and mechanism. J Clin Invest 1982;69:1286–92. 7085875 10.1172/JCI110568 PMC370201

[59] Gula LJ, Krahn AD, Skanes A, Ferguson KA, George C, Yee R, Heart rate variability in obstructive sleep apnea: a prospective study and frequency domain analysis. Ann Noninvasive Electrocardiol 2003;8:144–9. 12848796 10.1046/j.1542-474X.2003.08209.x PMC6932147

[60] Shi J, Piao J, Liu B, Pan Y, Gong Y, Deng X, Obstructive sleep apnea increases systolic and diastolic blood pressure variability in hypertensive patients. Blood Pres Monit 2017;22:208–12. 10.1097/MBP.0000000000000259 28394772

[61] Marrone O, Riccobono L, Salvaggio A, Mirabella A, Bonanno A, Bonsignore MR. Catecholamines and blood pressure in obstructive sleep apnea syndrome. Chest 1993;103:722–7. 8449058 10.1378/chest.103.3.722

[62] Sykora M, Diedler J, Rupp A, Turcani P, Steiner T. Impaired baroreceptor reflex sensitivity in acute stroke is associated with insular involvement, but not with carotid atherosclerosis. Stroke 2009;40:737–42. 19118245 10.1161/STROKEAHA.108.519967

[63] Colivicchi F, Bassi A, Santini M, Caltagirone C. Prognostic implications of right-sided insular damage, cardiac autonomic derangement, and arrhythmias after acute ischemic stroke. Stroke 2005;36:1710–5. 16020766 10.1161/01.STR.0000173400.19346.bd

[64] Oppenheimer S. Cerebrogenic cardiac arrhythmias: cortical lateralization and clinical significance. Clin Auton Res 2006;16:6–11. 16477489 10.1007/s10286-006-0276-0 PMC2782122

[65] Shoemaker J, Goswami R. Forebrain neurocircuitry associated with human reflex cardiovascular control. Front Physiol 2015;6. 10.3389/fphys.2015.00240 PMC4555962 26388780

[66] Oppenheimer SM, Wilson JX, Guiraudon C, Cechetto DF. Insular cortex stimulation produces lethal cardiac arrhythmias: a mechanism of sudden death? Brain Res 1991;550:115–21. 1888988 10.1016/0006-8993(91)90412-o

[67] Oppenheimer S, Cechetto D. The insular cortex and the regulation of cardiac function. Compr Physiol 2016;6:1081–133. 27065176 10.1002/cphy.c140076

[68] Kumar R, Farahvar S, Ogren JA, Macey PM, Thompson PM, Woo MA, Brain putamen volume changes in newly-diagnosed patients with obstructive sleep apnea. Neuroimage Clin 2014;4:383–91. 24567910 10.1016/j.nicl.2014.01.009 PMC3930100

[69] Macey PM, Wu P, Kumar R, Ogren JA, Richardson HL, Woo MA, Differential responses of the insular cortex gyri to autonomic challenges. Auton Neurosci 2012;168:72–81. 22342370 10.1016/j.autneu.2012.01.009 PMC4077282

[70] Pae EK, Chien P, Harper RM. Intermittent hypoxia damages cerebellar cortex and deep nuclei. Neurosci Lett 2005;375:123–8. 15670654 10.1016/j.neulet.2004.10.091

[71] George Zaki Ghali M. Midbrain control of breathing and blood pressure: the role of periaqueductal gray matter and mesencephalic collicular neuronal microcircuit oscillators. Eur J Neurosci 2020;52:3879–902. 32227408 10.1111/ejn.14727

[72] Hashimoto M, Yamanaka A, Kato S, Tanifuji M, Kobayashi K, Yaginuma H. Anatomical evidence for a direct projection from Purkinje cells in the mouse cerebellar vermis to medial parabrachial nucleus. Front Neural Circ 2018;12. 10.3389/fncir.2018.00006 PMC5808303 29467628

